# Assessing the asymptomatic reservoir and dihydroartemisinin–piperaquine effectiveness in a low transmission setting threatened by artemisinin resistant *Plasmodium falciparum*

**DOI:** 10.1186/s12936-016-1487-z

**Published:** 2016-09-01

**Authors:** Grégoire Falq, Rafael Van Den Bergh, Martin De Smet, William Etienne, Chea Nguon, Huy Rekol, Mallika Imwong, Arjen Dondorp, Jean-Marie Kindermans

**Affiliations:** 1 Médecins Sans Frontières, 46, rue de l’Arbre Bénit, 1050 Brussels, Belgium; 2 Médecins Sans Frontières, 68, rue de Gasperich, 1617 Luxembourg City, Luxembourg; 3 Centre for Parasitology, Entomology and Malaria Control, 477 Betong Street, Village Trapangsvay, Sanakat Phnom Penh Thmey, Khan Sen Sok, Phnom Penh, Cambodia; 4 Department of Molecular Tropical Medicine and Genetics, Faculty of Tropical Medicine, Mahidol University, 3/F, 60th Anniversary Chalermprakiat Building, 420/6 Rajvithi Road, Bangkok, 10400 Thailand; 5 Mahidol Oxford Tropical Research Unit, Faculty of Tropical Medicine, Mahidol University, Bangkok, Thailand

**Keywords:** Malaria, *Plasmodium falciparum*, Epidemiology, Artemisinin resistance, Polymerase chain reaction, *k13*-propeller, Cambodia

## Abstract

**Background:**

In Cambodia, elimination of artemisinin resistance through direct elimination of the *Plasmodium falciparum* parasite may be the only strategy. Prevalence and incidence at district and village levels were assessed in Chey Saen district, Preah Vihear province, North of Cambodia. Molecular and clinical indicators for artemisinin resistance were documented.

**Methods:**

A cross sectional prevalence survey was conducted at village level in the district of Chey Saen from September to October 2014. *Plasmodium* spp. was assessed with high volume quantitative real-time polymerase chain reaction (qPCR). *Plasmodium falciparum*-positive samples were screened for mutations in the *k13*-propeller domain gene. Treatment effectiveness was established after 28 days (D28) using the same qPCR technique. Data from the provincial surveillance system targeting symptomatic cases, supported by Médecins Sans Frontières (MSF), were used to assess incidence.

**Results:**

District *P. falciparum* prevalence was of 0.74 % [0.41; 1.21]; village prevalence ranged from 0 to 4.6 % [1.4; 10.5]. The annual incidence of *P. falciparum* was 16.8 cases per 1000 inhabitants in the district; village incidence ranged from 1.3 to 54.9 for 1000 inhabitants. Two geographical clusters with high number of cases were identified by both approaches. The marker for artemisinin resistance was found in six samples out of the 11 tested (55 %). 34.9 % of qPCR blood analysis of symptomatic patients were still positive at D28.

**Conclusions:**

The overall low prevalence of *P. falciparum* was confirmed in Chey Saen district in Cambodia, while there were important variations between villages. Symptomatic cases had a different pattern and were likely acquired outside the villages. It illustrates the importance of prevalence surveys in targeting interventions for elimination. Mutations in the *k13*-propeller domain gene (C580Y), conferring artemisinin resistance, were highly prevalent in both symptomatic and asymptomatic cases (realizing the absolute figures remain low). Asymptomatic individuals could be an additional reservoir for artemisinin resistance. The low effectiveness of dihydroartemisinin–piperaquine (DHA–PPQ) for symptomatic cases indicates that PPQ is no longer able to complement the reduced potency of DHA to treat falciparum malaria and highlights the need for an alternative first-line treatment.

## Background

Since 2000, huge efforts in malaria control have resulted in a 47 % decrease in malaria-associated mortality worldwide and in a 30 % decrease in incidence of malaria [1]. Unfortunately, these achievements are threatened by the emergence and spread of *Plasmodium falciparum* resistance to artemisinin and partner drugs. Current efforts to contain artemisinin resistance, consisting of a wide range of activities, such as long-lasting insecticide-treated net campaigns, implementation of accurate and widely-available malaria rapid diagnostic tests (RDT), banning of artemisinin monotherapies, and universal access to artemisinin-based combination therapy (ACT) [2], have yet to show success. Elimination of artemisinin resistance through direct elimination of the *P. falciparum* parasite may be the only present strategy [3].

In low transmission settings, the human asymptomatic and/or microparasitaemic reservoir is an important challenge in the context of malaria and artemisinin resistance elimination, as this reservoir typically escapes routine surveillance, but can contribute to active transmission [4, 5]. However, successful attempts to identify and quantify the asymptomatic reservoir have only rarely been documented.

Artemisinin resistance has been reported in Western Cambodia since 2008 [6–9]. Artemisinin resistance is defined as delayed parasite clearance, which represents a partial resistance. Recently, a molecular marker of artemisinin resistance was identified. Mutations in the Kelch 13 (k13)-propeller domain (especially C580Y) were shown to be associated with delayed parasite clearance in vitro and in vivo [10]. In 2013, Médecins Sans Frontières (MSF) in collaboration with the Ministry of Health initiated a programme for the elimination of *P. falciparum* in Chey Saen district, Preah Vihear province, in the North of Cambodia, a region with documented artemisinin resistance. This programme included two successive prevalence surveys (relying on molecular methods for the identification of asymptomatic carriers and of individuals carrying resistant parasite strains), and support and scale-up of the passive case detection network (relying on a strong collaboration with the public sector—Health Centers, Health Posts, Village Malaria Workers, and Malaria Contact Persons identified in important plantations and settlements—and the registered private sector in the district). Considering the dearth of information on the performance of surveys and routine surveillance in low malaria transmission settings, the objective of this study was (i) to document the prevalence and incidence of *Plasmodium* spp. and *P. falciparum* at district and village level in Chey Saen district, as assessed through respectively a prevalence survey and a passive case detection system (the aim was to be able to target villages with higher transmission and indicators of resistance), and (ii) to document the molecular and clinical indicators for artemisinin resistance in cases identified through both approaches.

## Methods

### Study setting

The study was conducted across all villages of the district of Chey Saen, Preah Vihear province, located in the North of Cambodia, bordering Thailand and the Lao People’s Democratic Republic. The district consisted of 21 villages, with two sub-villages considered as independent from the main village, resulting in 23 geographical units included in the study. The district population consisted of 22,343 individuals in 4585 households, and village sizes ranged from 328 to 2016 inhabitants (67–397 households). The study concerned the entire district population, but did not cover workers from a major Chinese company in the district, as access to this population was not possible.

### Study period

The prevalence study was conducted in September and October 2014. The study period for the passive case detection covered June 2014 to May 2015.

### Study population and sample size

For the prevalence survey, random sampling was done from the entire district population (a population census was done prior to the survey); children less than 1 year were excluded. Sample sizes were calculated per village, with an assumed *P. falciparum* prevalence of 7 %, a confidence of 95 %, a precision of 5 %, and a correction for finite populations based on each village size. If the target sample size for a village was not reached, a second sampling was done. To minimize absenteeism, participants were given an incentive for their participation (*krama*, a piece of local fabric).

For the passive case detection (PCD) component, all individuals presenting with fever and/or other malaria symptoms (headache, fatigue, body pain, nausea, etc.), in the public and private registered sector were included. Unregistered private providers were not included in the surveillance system. Private providers were mapped through consultative meetings with the village chiefs, community elders, and representatives of the private sector.

### Prevalence survey—methodology

For each participant of the prevalence survey, 3 ml of whole blood was drawn (0.5 ml for children less than 5 years of age) in EDTA tubes. Samples were centrifuged within 24 h to separate plasma and buffy coat, stored in a freezer (−20 °C), and transported to the Malaria Molecular laboratory at Mahidol University, Bangkok, Thailand, in cold chain. For each sample, high volume quantitative real-time PCR (qPCR) was performed, with a detection limit of 22 parasites per millilitre [11]. Samples were lysed in protease-containing buffer, and DNA extraction was performed using the QIAamp Blood Midi Kit (Qiagen). For samples containing parasite DNA, *Plasmodium* species detection was attempted using PCR protocols specific to detect the 18S rRNA gene of *Plasmodium* spp. Samples for which *Plasmodium* species could not be determined were reported as *Plasmodium* species. In each screening run, controls assessing both the DNA extraction and the PCR steps were included (extraction/PCR process, cross contamination and reagents).

*Plasmodium falciparum*-positive samples were screened for mutations in the k13-propeller domain gene, a molecular marker associated with artemisinin resistance [12]. Purified PCR products were sequenced at Macrogen, Republic of Korea (BioEdit v. 7.1.3.0., using the 3D7 kelch13 sequence as reference). The definition of single nucleotide polymorphisms (SNPs) was based on analytical approaches described previously [13, 14].

### Passive case detection—methodology

Case detection in the public—health facilities and village malaria workers (VMW)—and private sector was done by rapid test. The public sector used the First Response Malaria Ag. pLDH/HRP2 Combo Card Test (Premier Medical Corporation Limited, India till December 2014 and switched to SD BIOLINE Malaria Ag *P*.*f*/*P*.*v* (Standard Diagnostics, Korea) in 2015. The private sector usually relied on First Response Malaria or Malacheck (Standard Diagnostics, Korea). *P. falciparum*-positive cases (mono-infections and mixed infections) received dihydroartemisinin (DHA) and piperaquine (PPQ) (Eurartesim^®^, Sigma Tau, Italy) for 3 consecutive days (first dose under supervision, dosage according to national guidelines), and were tested for G6PD deficiency using the G6PD rapid test (G6PD Rapid Test CARESTART, Access Bio, USA). G6PD wild type patients were treated with a single dose of primaquine (0.25 mg/kg, REMEDICA, Cyprus). Patients were followed up 28 days (D28) after their initiation of treatment to establish treatment effectiveness, using the same qPCR technique as in the prevalence survey. D28 was chosen instead of D42 (traditional endpoint when using microscopy), to detect parasitological failures early enough and avoid transmission to the community of resistant parasites. Patients with D28 sustained parasitaemia were offered atovaquone-proguanil (Malarone^®^, GlaxoSmithKline, Belgium) as second-line treatment. G6PD screening, Primaquine treatment and D28 follow-up were implemented mid-December 2014.

A case investigation form (CIF) was completed by the MSF team for each confirmed malaria case (adapted from the WHO [15]). The CIF included demographic information and other characteristics, a history of the current illness including diagnostic test results and treatment, travel history, and possible mode of transmission.

Testing, DHA–PPQ treatment, and training on case management were provided by the public and private sectors. G6PD testing, primaquine treatment, and D28 follow-up were implemented by MSF teams. Health promotion messages were provided both by the public and private sectors and MSF teams.

### Statistical analysis

Prevalence calculations were conducted using sampling weights (W), calculated using village size (N) and number of respondents in each village (n) as follows: W = N/n. For prevalence at district level, the village was specified as sampling strata and a calibration adjustment was performed using a logit method [16]. The population census done in June 2014 was used and calibration variables were sex and age category (0–4, 5–14, 15–34, 35–65, 65–Inf). Considering the criteria for eligibility, the sample design and the calibration adjustment, the study population at the district level was estimated as representative of the population living in Chey Saen district. Confidence interval calculations used the scaled Chi squared distribution for the log likelihood from a binomial distribution [17]. District and village prevalence are presented with their 95 % confidence interval. Statistical analyses were conducted using R version 3.1.1 (R Development Core Team, 2014).

For PCD, the main outcomes were the monthly number of screened cases and confirmed *P. falciparum* (mono-infections and mixed infections) cases and the annual number of individuals treated per village, per month and per provider type. Incidences per village were calculated per year and per 1000 inhabitants using data from population census. Monthly incidences were calculated at district level.

### Ethical considerations

Individual written consent was obtained before inclusion in the prevalence survey and for all components of the MSF programme not included in the national guideline. Ethical approval was granted by the Cambodian National Ethics Committee for Health Research (280 NECHR) and the Ethics Review Board of Médecins Sans Frontières (ID1401).

## Results

### Prevalence survey—participation

**Fig. 1:**
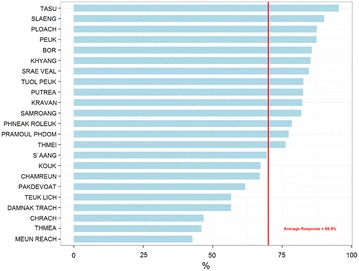
Response rate by village in the prevalence survey

**Fig. 2:**
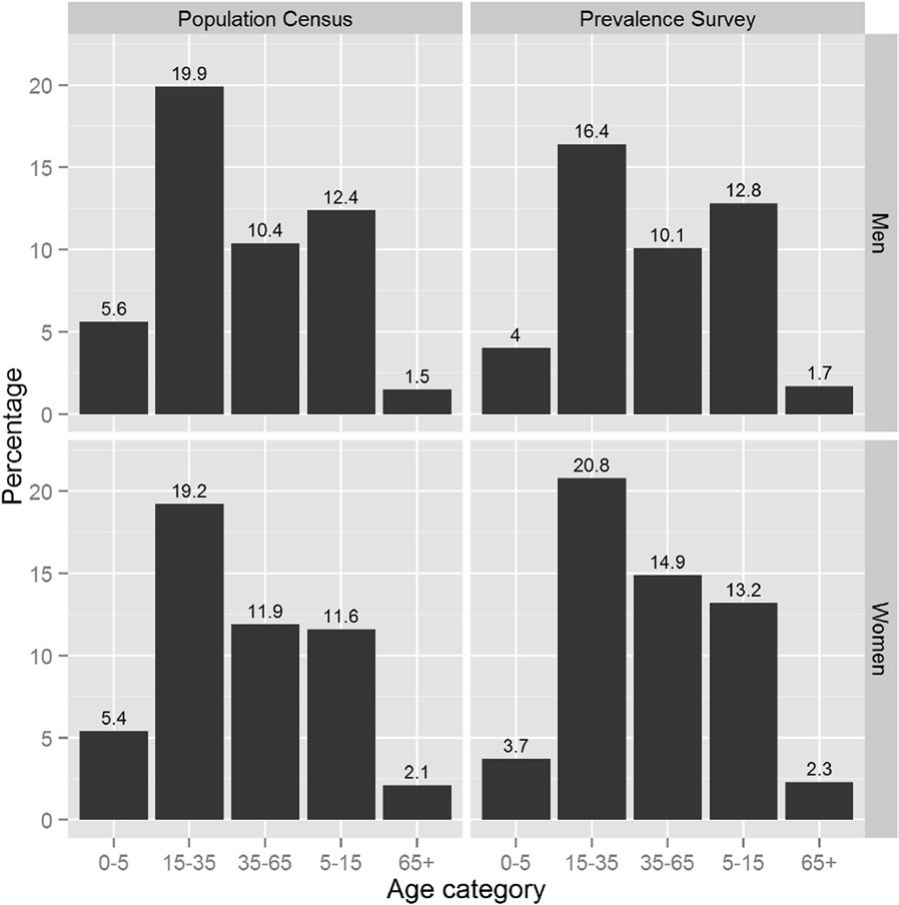
Proportion of district population and participant in prevalence survey by gender and by age category

Out of 2971 selected villagers, 2088 agreed and took part in the survey. The response rate per village ranged from 43 to 95 % (Fig. 1). At district level, males aged 15–34 years had the lowest (56 %) response rate, on the contrary women aged 15–34 years had the highest one (77 %). Reasons for non-response are summarized in Table 1, with “absence” (to the rice field, to the forest, travel, etc.) accounting for 53 % of the non-responses. Exclusion represented less than 5 %, and concerned mainly villagers who were too young or were sick at the moment of the survey. Overall, the age and gender distribution of the study participants was similar to that of the district population (Fig. 2), with mainly 1–4 year old respondents being under-represented (7.7 % in respondent vs 11.1 % in the district population).

**Table 1:** Reasons for non-response in the prevalence survey

	N	%
Absent	478	53.3
Excluded	37	4.1
Left after entry point	8	0.9
Moved	44	4.9
Name unknown	75	8.4
Reason not available	34	3.8
Refusal	210	23.4
Wrong location	11	1.2
Total	897	100.0

### Prevalence survey—results

**Fig. 3:**
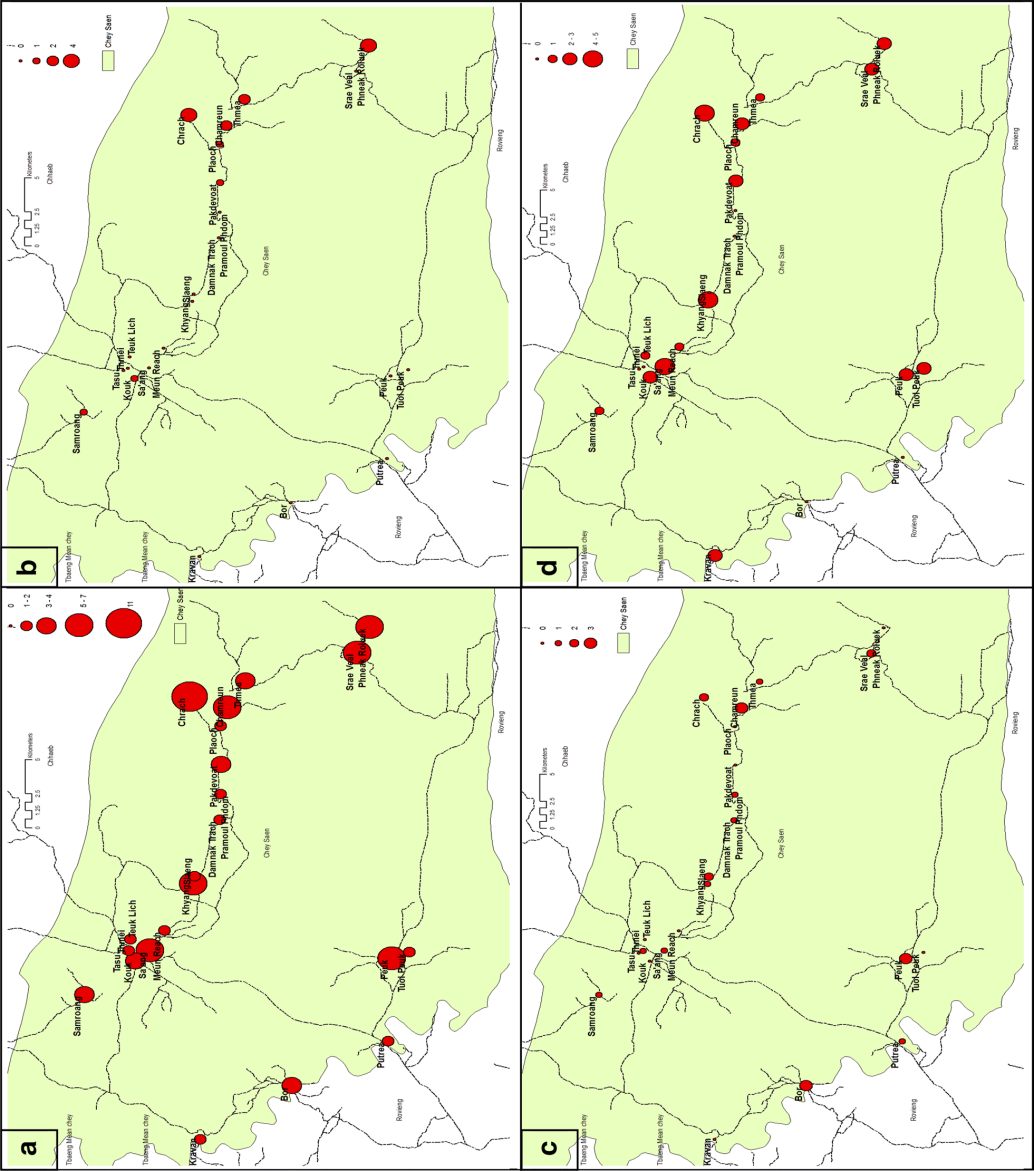
Geographic distribution of **a** all cases, **b** *Pf*-Mix cases, **c** *Pv* cases, **d** unspecified specie cases in the prevalence survey

Of the 2088 samples, four were coagulated before preparation and could not be analysed. 76 of the 2084 participants were screened positive as *Plasmodium* spp. carrier, resulting in a prevalence of 3.73 % [2.91; 4.68] after adjusting for village size (weighting); village prevalence ranged from 0 to 12.1 % [6.4; 20.0] (Table 2). No specific trend was found for the geographical distribution (Fig. 3). Five participants had fever (body temperature >37.5 °C) with a negative RDT. All positive cases were treated with a three-day course of DHA–PPQ and primaquine for G6PD wild type patients: parasitaemia at 28 days post-treatment was negative for all individuals.

**Table 2:** Prevalence, 95 % confidence interval by malaria species and by village in the prevalence survey

Village	*P. falciparum* ^a^		*P. vivax* ^a^		Unspecified		Total	
	Cases	Prevalence [CI 95]	Cases	Prevalence [CI 95]	Cases	Prevalence [CI 95]	Cases	Prevalence [CI 95]
Bor	0	0.0 [0.0; 0.0]	3	3.4 [0.8; 8.7]	0	0.0 [0.0; 0.0]	3	3.4 [0.8; 8.7]
Chamreun	2	2.4 [0.4; 7.4]	3	3.6 [0.9; 9.3]	2	2.4 [0.4; 7.4]	7	8.4 [3.6;15.9]
Chrach	4	4.4 [1.3;10.1]	3	3.3 [0.8; 8.5]	5	5.5 [1.9;11.6]	11	12.1 [6.4;20.0]
Damnak Trach	0	0.0 [0.0; 0.0]	1	1.2 [0.1; 5.4]	0	0.0 [0.0; 0.0]	1	1.2 [0.1; 5.4]
Khyang	0	0.0 [0.0; 0.0]	1	1.1 [0.1; 4.8]	4	4.3 [1.3;10.0]	5	5.4 [1.9;11.5]
Kouk	1	1.1 [0.1; 4.8]	0	0.0 [0.0; 0.0]	2	2.2 [0.3; 6.7]	3	3.3 [0.8; 8.4]
Kravan	0	0.0 [0.0; 0.0]	0	0.0 [0.0; 0.0]	2	2.3 [0.4; 7.0]	2	2.3 [0.4; 7.0]
Meun reach	0	0.0 [0.0; 0.0]	0	0.0 [0.0; 0.0]	1	1.0 [0.1; 4.6]	1	1.0 [0.1; 4.6]
Pakdevoat	1	1.1 [0.1; 5.1]	0	0.0 [0.0; 0.0]	2	2.3 [0.4; 7.1]	3	3.4 [0.8; 8.9]
Peuk	0	0.0 [0.0; 0.0]	3	3.1 [0.8; 8.0]	3	3.1 [0.8; 8.0]	6	6.3 [2.5;12.4]
Phneak Roleuk	4	4.6 [1.4;10.5]	1	1.1 [0.1; 5.1]	3	3.4 [0.8; 8.9]	7	8.0 [3.5;15.2]
Ploach	1	1.1 [0.1; 4.9]	0	0.0 [0.0; 0.0]	1	1.1 [0.1; 4.9]	2	2.2 [0.4; 6.9]
Pramoul Phdom	0	0.0 [0.0; 0.0]	1	1.2 [0.1; 5.2]	0	0.0 [0.0; 0.0]	1	1.2 [0.1; 5.2]
Putrea	0	0.0 [0.0; 0.0]	1	1.1 [0.1; 4.7]	0	0.0 [0.0; 0.0]	1	1.1 [0.1; 4.7]
S’aang	0	0.0 [0.0; 0.0]	1	1.0 [0.1; 4.5]	4	4.1 [1.2; 9.4]	5	5.1 [1.8;10.8]
Samroang	1	1.1 [0.1; 4.9]	1	1.1 [0.1; 4.9]	1	1.1 [0.1; 4.9]	3	3.3 [0.8; 8.6]
Slaeng	0	0.0 [0.0; 0.0]	2	2.0 [0.3; 6.2]	0	0.0 [0.0; 0.0]	2	2.0 [0.3; 6.2]
Srae veal	0	0.0 [0.0; 0.0]	2	2.3 [0.4; 7.0]	3	3.4 [0.8; 8.8]	5	5.7 [2.0;12.0]
Tasu	0	0.0 [0.0; 0.0]	0	0.0 [0.0; 0.0]	0	0.0 [0.0; 0.0]	0	0.0 [0.0; 0.0]
Teuk Lich	0	0.0 [0.0; 0.0]	0	0.0 [0.0; 0.0]	1	1.2 [0.1; 5.4]	1	1.2 [0.1; 5.4]
Thmea	2	2.1 [0.3; 6.5]	2	2.1 [0.3; 6.5]	1	1.1 [0.1; 4.7]	4	4.2 [1.3; 9.7]
Thmei	0	0.0 [0.0; 0.0]	1	1.3 [0.1; 5.6]	0	0.0 [0.0; 0.0]	1	1.3 [0.1; 5.6]
Tuol Peuk	0	0.0 [0.0; 0.0]	0	0.0 [0.0; 0.0]	2	2.1 [0.3; 6.5]	2	2.1 [0.3; 6.5]
District	16	0.74 [0.41; 1.21]	26	1.33 [0.85; 1.97]	37	1.84 [1.28; 2.54]	76	3.73 [2.91; 4.68]

^a^Including mixed infections of *P. falciparum* and *P. vivax*

Following species determination, *P. falciparum* (mono and mixed infections) was identified in 16 participants, giving a prevalence of 0.74 % [0.41; 1.21] after weighting the villages. Village prevalence ranged from 0 to 4.6 % [1.4; 10.5] (Table 2). Median parasitaemia was 30,142 p/ml (IQR: 578,839). Six of the eight villages with presence of *P. falciparum* were located in the East of the district, and five of them were in the same area and were geographically connected (Fig. 3). Seventy five percent (8/12) of *P. falciparum* carriers identified were male. *Plasmodium falciparum* was not found in the age groups of 0–4 years and 65+ years; the 15–34 age group accounted for a half of the total number of cases (Table 3).

**Table 3:** Number and percentage of cases by species and by gender and age category in the prevalence survey

	Female		Male		Total	
	N	%	N	%	N	%
*P. falciparum* ^a^						
0–4	0	0.0	0	0.0	0	0.0
5–14	1	6.3	1	6.3	2	12.5
15–34	3	18.8	5	31.3	8	50.0
35–64	0	0.0	6	37.5	6	37.5
>64	0	0.0	0	0.0	0	0.0
Total	4	25.0	12	75.0	16	100.0
*P. vivax* ^a^						
0–4	1	3.8	0	0.0	1	3.8
5–14	2	7.7	2	7.7	4	15.4
15–34	3	11.5	11	42.3	14	53.8
35–64	0	0.0	6	23.1	6	23.1
>64	0	0.0	1	3.8	1	3.8
Total	6	23.1	20	76.9	26	100.0
*P.* unspecified						
0–4	0	0.0	0	0.0	0	0.0
5–14	1	2.7	3	8.1	4	10.8
15–34	5	13.5	13	35.1	18	48.6
35–64	7	18.9	6	16.2	13	35.1
>64	2	5.4	0	0.0	2	5.4
Total	15	40.5	22	59.5	37	100.0
All species						
0–4	1	1.3	0	0.0	1	1.3
5–14	4	5.3	6	7.9	10	13.2
15–34	11	14.5	27	35.5	38	50.0
35–64	7	9.2	17	22.4	24	31.6
>64	2	2.6	1	1.3	3	3.9
Total	25	32.9	51	67.1	76	100.0

^a^Including mixed infections of *P. falciparum* and *P. vivax*

*Plasmodium vivax* (mono and mixed infections) was identified in 26 participants, giving a prevalence of 1.33 % [0.85; 1.97], and ranging from 0 to 3.6 % [0.9; 9.3] between villages (Table 2); no geographical pattern was observed (Fig. 3). More than 75 % (20/26) of *P. vivax* carriers identified were male. *P. vivax* was found in all age groups, with more than a half in the 15–34 years age group. In 37 *Plasmodium* spp. carriers (48.7 %), species determination could not be conducted due to insufficient genetic material (too low parasitaemia).

Of the 16 *P. falciparum* samples, in 11 sufficient *k13* gene was amplified by PCR for subsequent sequencing. The mutant-type allele (C580Y) was found in six samples (55 %). This indication of resistance to artemisinin was found in the Eastern (Chamreun, Chrach, Ploach and Phneak Roleuk) and Western (Kouk and Samroang) parts of the district.

### Passive case detection

**Fig. 4:**
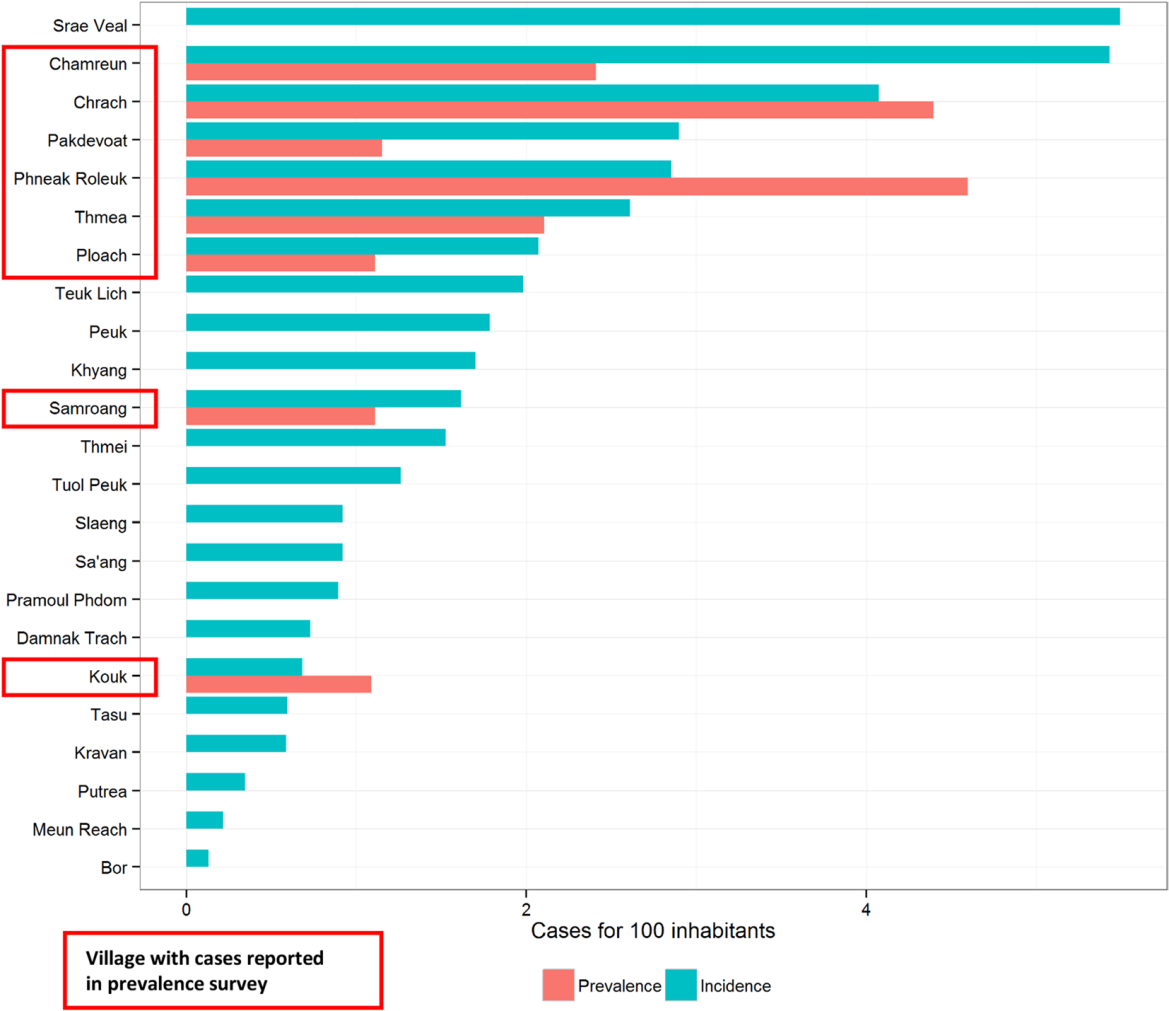
Comparison of *P. falciparum* incidence and prevalence per village

At district level, 3007 villagers (13.5 % of the population) were screened for malaria during the study period (Table 4). Most screening was provided by village malaria workers (2269; 75.5 %), followed by health facilities (397; 13.3 %) and registered private providers (290; 9.6 %). The surveillance system identified 157 cases of *P. falciparum* mono-infection and 213 cases of mixed infection. The total of *P. falciparum* (mono-infections plus mixed infections) was 370 cases. This gave an annual incidence of *P. falciparum* of 16.8 cases per 1000 inhabitants in the district. Village incidence ranged from 1.3/1000 inhabitants in Bor to 54.9/1000 inhabitants in Srae Veal (Fig. 4). Seven villages had an incidence higher than 20/1000 inhabitants: all were located in the Eastern part of the district and could be considered as two geographically connected clusters. The 1st cluster included Chamreun (Incidence = 54.3), Chrach (I = 40.7), Pakdevoat (I = 27.5), Thmea (I = 26.1) and Ploach (I = 20.7). The 2nd cluster included Srae Veal (I = 54.9) and Pheak Roleuk (I = 28.5). Most *P. falciparum* cases were male, and more than half of all cases belonged to the 15–34 years age group (Table 5).

**Table 4:** Number of villagers screened and positivity rate per provider type

Provider	Screened		Pf	Positivity rate
	N	%		
Health center	188	6.3	50	26.6
Health post	209	7.0	36	17.2
MSF	50	1.7	4	8.0
Private	290	9.6	49	16.9
VMW	2269	75.5	231	10.2
Total	3007	100.0	370	12.3

**Table 5:** Number of *Plasmodium falciparum* cases by gender and by age category in PCD

	Female		Male		Total	
	N	%	N	%	N	%
0–4	3	0.8	4	1.1	7	1.9
5–14	12	3.2	31	8.4	43	11.6
15–34	11	3.0	201	54.3	212	57.3
35–64	23	6.2	79	21.4	102	27.6
>64	3	0.8	3	0.8	6	1.6
All	52	14.1	318	85.9	370	100.0

**Fig. 5:**
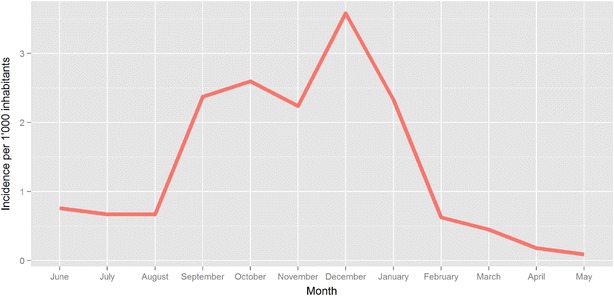
Monthly incidence for *P. falciparum*

Analysis of monthly incidence identified a high transmission season from September to January. June to August incidence was around 0.7/1000 per month, September to December incidence ranged from 2.2 to 3.6/1000 per month, and February to May incidence ranged from 0.2 to 0.6/1000 per month (Fig. 5). 149 villagers had blood taken 28 days after case notification, to establish treatment effectiveness. 52 of these samples (34.9 %) were still positive at day 28 by qPCR (Table 6).

**Table 6:** D28 results in PCD and prevalence survey

	Negative		Positive		Total	
	N	%	N	%	N	%
November	10	35.7	18	64.3	28	100
December	40	72.7	15	27.3	55	100
January	38	79.2	10	20.8	48	100
February	6	50.0	6	50.0	12	100
March	3	50.0	3	50.0	6	100
All	97	65.1	52	34.9	149	100
Prevalence survey	48	100	0	0.0	0	0

223 CIF of *P. falciparum* (mono and mixed infections) cases were available for analysis. The occupation of most cases was farmer (175, 78.5 %); most spent at least one night in forest, field or plantation the month preceding blood sampling (197, 88.3 %). Almost 50 % of these forests, fields and plantations were located in the Eastern part of the district (areas with high prevalence and incidence) around the villages of Thmea (41 cases, 21.8 %) and Chrach (32 cases, 16.2 %), and the bordering Stung Treng province (22 cases, 11.2 %).

## Discussion

The primary objectives of this study were to estimate the prevalence of *Plasmodium* spp. and *P. falciparum* at district and village level in Chey Saen district and to compare this with the incidence reported through the passive case detection system. At the same period of the year (September and October), the prevalence of *Plasmodium* spp. was slightly elevated (from 2.61 to 3.73) compared to the findings of a survey conducted in 2013 [18].

Despite the fact that the current survey relied on PCR testing of high blood volume, which is more sensitive than PCR from a filter paper dried blood spot (volume around 5 µl) as used in the 2013 survey, results showed a decrease at district level in the prevalence of *P. falciparum* (with a slight overlap of confidence intervals). *Plasmodium falciparum* prevalence remains low in Chey Saen district. Similar low prevalence and variation in the dynamic of transmission have also been observed, using this sensitive molecular method. For instance in Pailin [18], the prevalence of *P. falciparum* dropped from about 2 % in June 2013 to much lower than 1 % in June 2014. [19]. In addition, MSF programme has been running in 2014, focusing on *P. falciparum* cases management. The decrease in the ratio *P. falciparum*/*P. vivax* is typical in areas of pre-elimination when treatment programmes (with ACT) are correctly functioning for each malaria episode, without implementing a radical cure for patients with persistent *P. vivax* infections.

The study had a number of limitations. The prevalence survey was designed to estimate prevalence at village level and to capture all parasite carriers from a representative sample, but a bias may have been introduced due to preferential absenteeism of the male young adult population (the most at risk sub-population). However, as characteristics of respondents in terms of gender and age category were close to district population, and age and gender-adjustment was performed, a sizeable underestimation of the true district prevalence was not anticipated. Likewise, while temporary workers living in the compound of private companies (i.e. plantations) were not reachable, which may have led to an underestimation of the prevalence, this specific group was probably limited in size.

Overall, passive case detection and prevalence survey showed very similar results to the extent that the two geographical clusters with high number of cases were identified by both approaches. Six out of the seven villages with higher incidence were also identified through the prevalence survey, suggesting that villages with high incidence also had a sizeable asymptomatic reservoir. However, the prevalence survey failed to identify *P. falciparum* cases in 15 villages where cases were detected passively, and the village with highest malaria incidence had no positive cases in the prevalence survey. This may be the consequence of the survey design, as it was not powered for detection of low prevalence at village level as well as areas with specific and potentially limited in size at risk populations. Forest goers are also more likely to be out of the village during the survey. If parasite prevalence is indeed a better surveillance measure for elimination programs than numbers of symptomatic cases [20], surveys have to enroll high numbers of participants per geographical unit studied. This may render the monitoring of elimination interventions highly resource-demanding in low prevalence settings. Passive and active case detection (focusing on at-risk populations) may thus remain an important tool to monitor malaria elimination.

Results can suggest that transmission in the villages is limited. Discrepancies between incidence and prevalence might be partly explained by the absence of infected people during the prevalence survey. Those who stay more permanently in the villages (hence more often included in the survey) would be less likely to be positive. In addition, prevalence of asymptomatic cases is either not detected in 15 villages, or very low in the seven others. Symptomatic patients may have been infected elsewhere (e.g. in the forest) and not through local transmission in the villages.

The distribution of positive *P. falciparum* cases per age group also suggests that infection occurs outside the villages: cases passively detected in the 0–4 age group represent 1.9 % of total cases (and 0 % in the prevalence survey), while children under five account for 11 % of the overall population. Young children are staying most of the time inside the villages, as they are indeed less susceptible to accompany adults outside. Finally the fact that 85.9 % of *P. falciparum* cases were men (and 54.3 % in the 15–34 age group), is also an indication of limited village transmission: women are well known to stay much more inside the village (despite not to the extent of young children). This possible transmission pattern outside of the villages should be confirmed with further studies as this has a direct impact on elimination strategies, particularly on vector control.

Amongst the 16 *P. falciparum* positive samples of this survey, only 11 could be screened for mutations (indicating artemisinin resistance), and six (55 %) presented the mutant-type *k13* allele (C580Y) closely associated with artemisinin resistance. Asymptomatic individuals could thus be an additional reservoir for artemisinin resistance. Cases were found both in Eastern and Western part of the district, which means that *k13* resistance-associated mutations are not confined to a limited area. This result was in accordance with the findings of the 2013 survey (seven *P. falciparum* samples out of 11–64 %-presented the mutant-type *k13* allele), and suggests artemisinin resistance is a public health threat in Chey Saen district (realizing the absolute figures remain low). An additional reason for concern is the analysis of the treatment efficacy for symptomatic cases, with more than a third of all cases still positive with PCR 28 days after administration of first-line treatment. While poor treatment adherence and poor drug quality/storage conditions cannot be excluded in this study, recent drug efficacy studies using the standard WHO therapeutic efficacy study protocol for symptomatic cases have recently observed similar high treatment failure rates in two provinces bordering Preah Vihear province, with an adequate clinical and parasitological response at D42 in Siem Reap of 37.5 % and in Stung Treng of 66.7 % (Results from ‘TES and K13 Surveillance’, CNM, presentation at Malaria Elimination Partners Convening—May 2015).

Overall these results highlight the low effectiveness of DHA–PPQ as first-line treatment in many parts of Cambodia, and that resistance/treatment failure has been moving from the ‘classical’ Western Thai-Cambodian border area towards the Northern area. The findings of this study indicate that DHA–PPQ may no longer be effective for symptomatic cases with high parasitaemia. This may be due to resistance to artemisinin, as well as to the piperaquine partner drug [21–24]. On the other hand, D28 on asymptomatic cases identified through the prevalence survey were all undetectable, suggesting that clearance of low parasitaemias in asymptomatic carriers was still achieved with this drug combination. To demonstrate however full efficacy on asymptomatic carriers, a longer follow up would be necessary in a larger sample size. This is important for the choice of anti-malarial treatment in mass drug administrations, aimed to target the asymptomatic reservoir.

These data illustrate the importance of using highly sensitive diagnostic tools to identify the asymptomatic carriers. They invite to take action against this reservoir, which contribute to maintaining transmission.

They also show big variations between villages, justifying different type of interventions (in addition to good traditional control programmes) depending on the levels of prevalence and incidence: active case detection aiming at profiling and targeting at risk people, reactive case detection (whose positivity rate is higher than passive case detection in the area), or targeted malaria treatment with mass drug administration proposed to certain groups.

## Conclusions

The overall low prevalence of *Plasmodium* spp. and *P. falciparum* in Chey Saen district in Cambodia was confirmed by the prevalence survey, while there were important variations between villages. Symptomatic cases had a different pattern and were likely acquired outside the villages. It illustrates the importance of prevalence surveys in targeting interventions for elimination. Mutations in the *k13*-propeller domain gene (C580Y), conferring artemisinin resistance, were highly prevalent in both symptomatic and asymptomatic cases, indicating its significance in Chey Saen district (realizing the absolute figures remain low). Asymptomatic individuals could be an additional reservoir for artemisinin resistance. The low effectiveness of DHA–PPQ for symptomatic cases indicates that PPQ is no longer able to complement the reduced potency of DHA to treat *falciparum* malaria and highlights the need for an alternative first-line treatment.

## Abbreviations

PCR: polymerase chain reaction
DHA–PPQ: dihydroartemisinin–piperaquine
RDT: rapid diagnostic test
ACT: artemisinin-based combination therapy
PCD: passive case detection
EDTA: ethylenediamine tetraacetic acid
DNA: deoxyribonucleic acid
RNA: ribonucleic acid
pLDH/HRP2: parasite lactate dehydrogenase/histidine-rich protein-2
G6PD: glucose-6-phosphate dehydrogenase deficiency
CIF: case investigation form
WHO: World Health Organization
VMW: village malaria workers
